# The Inter-ministerial National Structural Intervention trial (INSTRUCT): protocol for a parallel group cluster randomised controlled trial of a structural intervention to reduce HIV infection among young women in Botswana

**DOI:** 10.1186/s12913-018-3638-0

**Published:** 2018-10-30

**Authors:** Anne Cockcroft, Nobantu Marokoane, Leagajang Kgakole, Joseph Kefas, Neil Andersson

**Affiliations:** 1 Department of Family Medicine, McGill University, Montreal, Canada; 2 CIET Trust Botswana, PO Box 1240, Gaborone, Botswana; 3 National AIDS Coordinating Agency, Gaborone, Botswana; 4 Centro de Investigación de Enfermedades Tropicales, Universidad Autónoma de Guerrero, Acapulco, Mexico

**Keywords:** HIV prevention, Structural intervention, Young women, Choice disability, Cluster randomised controlled trial, Botswana

## Abstract

**Background:**

Wide recognition that structural factors are important in the HIV epidemic has not generated much evidence of impact of structural interventions. Few randomised controlled trials of structural interventions for HIV prevention have an HIV endpoint, and most of those did not show a significant impact. It has proved difficult to prevent new HIV infections in Botswana, especially among young women, many of whom are unable to act on HIV preventive choices. Proposed by a government think tank in Botswana, the Inter-ministerial National Structural Intervention trial (INSTRUCT) tests whether addressing social and economic factors, including gender inequality, gender violence, poverty, and poor access to education, can lower HIV infection rates among young women. Focussed on increasing access by marginalised young women to government support programs, the intervention seeks to change their structural position, reducing their vulnerability to transactional sex, and thus to HIV infection.

**Methods:**

This parallel group cluster randomised controlled trial compares HIV rates among young women in districts with and without the structural interventions. The 30 administrative districts in the country, stratified by HIV prevalence and development status, will be randomly assigned to 5-district implementation waves. The intervention in the first-wave districts will include: (i) recruiting and preparing vulnerable young women to apply to government support programs, (ii) making the support programs more accessible to young women by engaging local program officers and young women in co-evaluation of programs and co-design of solutions; and (iii) generating an enabling environment for change in communities through an audio-drama edutainment program. In year five, an impact survey will measure HIV rates among vulnerable young women (15–29 years) in a random sample of communities in the five intervention districts and in the five second-wave (control) districts. Fieldworkers will undertake rapid HIV screening and interview young women and young men, collecting information on secondary outcomes of attitudes and behaviours.

**Discussion:**

This is the first step in a planned stepped-wedge design that will roll out the intervention, modified as necessary, to all districts. Strong government commitment provides an important opportunity to reduce new HIV cases in Botswana, and guide prevention efforts in other countries.

**Trial registration:**

Registration number: ISRCTN 54878784. Registry: ISRCTN. Date of registration: 11 June 2013.

## Background

Structural factors like poverty, poor education, gender inequality, and gender violence are important in the continuing HIV epidemic, and nowhere more so than in the southern Africa epicentre [1–7]. Many people are constrained in protecting themselves against HIV; this choice disability [8] is particularly common among young women in Botswana, who continue to bear the brunt of new infections [9, 10].

Despite increasing recognition of the role of structural factors in the HIV epidemic [9, 11, 12], relatively few randomised controlled trials (RCT) have tested the impact of structural interventions for HIV prevention, and even fewer have had a biological (HIV infection) endpoint. Among 10 trials reported [13–22], only three reported a significant impact on HIV status or incidence [20–22]. Most of the trials applied quite small interventions at individual level, like cash payments conditional on certain behaviours (such as remaining in school) or micro-credit schemes. These were not truly structural in the sense of changing the objective position of young women in their society, at least not in a way that helped them to act on their prevention choices.

A cluster RCT in 77 communities in Botswana, Namibia and Swaziland in 2008–2012 tested the impact on HIV rates in young women of a complex intervention to reduce choice disability (ISRCTN28557578). The intervention was associated with a reduction in HIV rates in Botswana, not found in the other two countries (N Andersson, personal communication). One possible reason for the greater impact in Botswana may have been the greater opportunities for young women to benefit from government structural support programs in Botswana than elsewhere.

Botswana spends a sizeable proportion of its gross domestic product (GDP) on social protection (4.4%), the bulk of it on the social safety net and a lesser proportion on labour market programs [23]. The latter aim to improve employability through education, skill building, support for livelihood initiatives, and obtaining work experience through community service placements. Access to these and other government structural support programs could change the objective position of marginalized young women, increase their social participation, generate greater (financial) independence, increase their sense of self-worth, and thus decrease their choice disability.

These government structural support programs were not designed to benefit young women or to prevent HIV. Use by marginalised young women is low: in a recent study among young women aged 18–29 years who were not in school and not in work, only 33% of young women had ever applied and 10% had ever benefitted from any program, excluding a part-time minimum wage rotating employment scheme with no training or development elements. More educated young women were more likely to be accepted into these programs, indicating a double disadvantage for those who had dropped out of school, for example due to unplanned pregnancies [24].

The Inter-ministerial National Structural Intervention trial (INSTRUCT) arose from discussions in the Botswana HIV Prevention Think Tank, a high-level government advisory body convened by the National AIDS Coordinating Agency (NACA). Recognising the dearth of robust evidence of the impact of structural interventions for HIV prevention, NACA and CIET proposed a national cluster randomised controlled trial of a structural intervention.

The research question is: Can a structural intervention, applied at district level to increase the access of marginalized young women to government structural support programs and provide an enabling environment for their emancipation, reduce HIV infections among these young women?

### General objective

Reduce the number of new HIV infections in Botswana, particularly among young women, who bear the brunt of these infections.

### Specific objectives

1. Implement, in five randomly selected districts, a composite structural intervention: to build skills and support young women to apply to available government support programs; to make support programs more accessible to young women; and to create an enabling environment for young women to exercise protective choices.
2. Measure the impact of the intervention on HIV rates in marginalized young women aged 15–29 years living in intervention districts, compared with young women living in five randomly selected control districts.
3. Measure the impact of the intervention on intermediate outcomes in a behaviour change model in young women and young men, comparing intervention and control districts.
4. Document costs of the intervention package in relation to its outcomes, to calculate costs per case averted and confront the cost implications of its wider roll-out.

A broader outcome, if the trial indicates impact on HIV rates, will be to stimulate further trials of structural interventions appropriate to the context with HIV infections as an endpoint. This will be relevant in Southern Africa and in other regions. In the meantime, the findings from Botswana should make a case for scaling up the interventions in that country.

### Theory of change

In the INSTRUCT trial, HIV-vulnerable young women are 15–29 years of age, not in school and not in work. We are especially concerned about those “kwa masimo” -- typically pregnant early teens who are sent to live and to work on family agricultural lands outside the main villages. The government of Botswana has in place support programs to help people move out of poverty, improve their education, and improve their livelihoods through small enterprises, but these are rarely accessed by young women [24].

We believe effective access to support programs by vulnerable young women will: (i) decrease the need for young women to engage in inequitable transactional sex with its attendant risks of unanticipated pregnancy and sexually transmitted infections (including HIV) for them and their subsequent sexual partners [25]; (ii) reduce educational and economic disparities with re-inclusion of marginalised young women, with the human rights implicit in this inclusiveness making for a healthier and more peaceful society [26]; (iii) make available disposable income at the very bottom of Botswana’s socio-economic pyramid, which will help to reduce chronic hunger, cut extreme poverty and boost local economic development as this investment finds its way into food and clothing purchases, rents and transport [27]. Many vulnerable young women in Botswana are single unemployed mothers, so access to support programs can benefit entire families as they spend new incomes to benefit their children, improving their nutrition, health and educational opportunities.

Compounding the HIV risks for young women is a “culture of violence” that affects everyone in communities where gender violence is common – girls, boys, young women and young men, older women and older men [28]. The *Beyond Victims and Villains* audio-drama in the INSTRUCT intervention will confront this culture of violence in communities and schools. As this culture is questioned or redressed, we expect a short-term reduction in gender violence and a shift in the culture of violence sustaining it [29]. Based on a considerable body of evidence [30–32], we expect the reduced gender violence will improve mental health and well-being of women and girls directly, as well as reducing subsequent high-risk behaviours for HIV, other sexually transmitted diseases, and unanticipated pregnancy. We further assume the reduction in gender violence will also benefit boys and young men; up to the age of 14 years, boys in southern Africa suffer rates of sexual abuse as high as those in girls [33, 34]. People abused as children are more likely to grow up to be abusers, so reducing child abuse will have wider than individual benefits [35, 36].

### Trial design

This trial is intended to be the first phase of a proposed, larger stepped-wedge trial to roll out an approach to increasing access of marginalised young women in Botswana to government structural support programs, measuring, among other outcomes, the impact on HIV rates [37]. This initial parallel group cluster randomised controlled trial will compare five randomly selected districts, in the first wave of the stepped-wedge, with five randomly selected districts in the second wave of the stepped-wedge as control districts.

## Methods

### Study setting

Botswana, in Southern Africa, is roughly the size of France, with a population of about two million, mainly concentrated in the South East around the capital, Gaborone. The adult (15–49 years) HIV prevalence in Botswana at 21.9% in 2016 is amongst the highest in the world [38]. Women are disproportionately affected, with an adult prevalence of 26.3%, compared with 17.6% among men [38]. Despite prevention efforts, the incidence of new HIV infections in Botswana has been resistant: the 2013 Botswana AIDS Impact Survey (BAIS IV) reported an (uncorrected) HIV annual incidence of 2.92% in the population aged 18 months to 64 years, the same as the equivalent figure in the 2008 BAIS III survey [39]. The BAIS IV “raw” incidence figures for both 2008 and 2013 are over-estimates of incidence but they illustrate the lack of important reduction in incidence. The 2016 UNAIDS estimate for HIV incidence in adults aged 15–49 in Botswana is 9.32 per 1000 [38].

The Botswana government provides a range of programs offering grants or loans, intended to help people improve their educational qualifications, start small enterprises, build skills to increase employability, and improve livelihoods. Young women and men of 18 years and above are eligible for most of these programs, and some specifically target youth. Up to eight ministries are involved in planning and managing these programs. Programs include support for growing crops or keeping livestock, support to return to school or use distance learning, a youth apprenticeship scheme, a scheme to support youth enterprises, various schemes for those below a poverty line, training and support for entrepreneurship, and a rotating minimum wage part-time employment scheme, *Ipelegeng*, which does not provide any training or development support for participants, but is easy to apply for and readily available, especially in more remote areas.

### Participants and eligibility criteria

#### Inclusion criteria

##### For intervention

All 30 districts are eligible for randomization to receive the intervention. In each intervention district, the different intervention activities will address all members of different groups of the population, who will be free to decide whether to take up the offer. Workshops to build skills and self-confidence, and to help young women apply for government support programs will be offered to women aged 18–25 years, not in school and not in work, in all communities in each district. Educational audio-drama discussion groups will be offered to people of all ages and both sexes in each community, including school students. Program officers and managers of all government support programs in the district will be invited to discussions about the obstacles for young women accessing their services and how these could be overcome.

##### For impact surveys

Young women and young men aged 15–29 years in 10 clusters (random selection of enumeration areas from the recent census) in each of five intervention and five non-intervention districts.

#### Exclusion criteria

##### For intervention

No district will be excluded. Within each intervention district, people not resident in the district will be excluded to minimize possible contamination (by people in other districts hearing of the interventions and wishing to take advantage of them, thus diluting the measured impact).

##### For impact surveys

Field workers will not interview or take blood samples from anyone they judge to be unable to give informed consent or to understand the survey questions.

### The interventions

INSTRUCT will use a triad of interventions to change the objective position of the most marginalised young women in Botswana: helping young women to access government programs, to change their position in their communities, and to make choices in their lives, including HIV prevention choices; working with existing government support programs to make them more accessible for young women; and creating an enabling community environment through audio-drama discussion groups.

#### Focused workshops (FW) for young women

This intervention combines advice and training for young women with helping them to take advantage of existing government support programs. It aims to increase self-esteem and assertiveness, as well as provide access to income-generating support programs, so transforming HIV risk. District INSTRUCT field coordinators in each intervention district, trained by the central team of INSTRUCT investigators, will implement FW in each community in the district. The District coordinators will try to identify all young women aged 15–29 in each community who are not in school and not in work. We have developed a method – respondent-driven recruitment – to do this, derived from respondent-driven sampling. Local field workers will identify and interview eligible young women by going door to door, by asking eligible young women to direct them to other eligible young women, and by seeking the advice of community leaders, social workers and health education assistants in each community. Our field workers will be young women from the district, and their knowledge will be important in identifying eligible participants.

They will interview these young women and invite those aged 18–25 years to attend a two-day workshop. Each workshop will have about 20–25 participants, and the number of workshops required in each community will vary. The interactive workshop will cover skills of communication, negotiation, resisting peer pressure, and building assertiveness and self-esteem. On the second day of the workshop, local program officers from government support programs will attend to talk about their programs and answer questions about how to apply. The workshop intervention will not itself provide capital or loans; these will come from the existing programs. The district coordinators will follow up young women participants after the workshops to check their progress and help to resolve difficulties with access to programs.

#### Making government support programs more accessible for young women

In each intervention district, INSTRUCT investigators will interview program managers and officers of government support programs available to young women to discuss with them the evidence that young women rarely access these programs and hear their views about why this might be and what could be done to improve accessibility.

*Fuzzy cognitive mapping* (FCM) is a participatory technique for different stakeholders to create a visual display of their knowledge and beliefs about what causes a given outcome [40, 41]. In this case, the stakeholder maps will show their views and their experience of obstacles and facilitators to access government support programs by marginalized young women. Two groups of stakeholders will create maps in each intervention district: marginalized young women and program officers of government support programs. We will recruit young women from among those attending focused workshops (above) to attend half-day workshops for creating cognitive maps. Our experience with cognitive mapping in rural Botswana and other resource-poor settings confirms that young women, even with limited literacy, can create very informative maps and their experience doing so sparks their interest and gives them confidence to discuss workable solutions. This prepares them to participate in deliberative dialogue sessions with service providers. Local program officers bring to the mapping their detailed expertise to identify bottlenecks and obstacles to successful applications, in this exercise focusing their concern on young women. This not only maps obstacles and opportunities for young women, but it makes the service providers aware of the special issues of vulnerable young women. With the support of line ministries and the District Commissioner in each of the five districts, we will identify program officers from all relevant government programs, inviting them to the half-day participatory fuzzy cognitive mapping exercise.

*Deliberative dialogue* will involve both service providers and young women who are authors of the fuzzy cognitive maps. Deliberative dialogue begins with evidence and finishes with a list of recommendations, without necessarily achieving consensus. An obvious risk is that education and gendered power disparities between service providers and young women can silence the voices of the young women. Piloting deliberative dialogue, we developed several procedures to increase the chances of real and productive dialogue and co-design. First, we will recruit the most articulate and outspoken of the young women attending focused workshops and cognitive mapping sessions to join the deliberative dialogue sessions. Second, before the open forum towards the end of the dialogue looking for common ground, we will provide an opportunity for the young women participants to present their own map and to comment on the similarities and differences with the service providers’ map. Third, as the dialogue progresses, the facilitator will open a space for comments by the young women if they are getting less chance to talk than they need. It is likely that deliberative dialogue will identify and provide some sort of hierarchy – at very least divided into quick turn-arounds and longer-term system fixes – of the issues that need to be changed to increase the chances of success of young women applying to programs, as well as ensuring that programs provide support for the most marginalised young women to apply. We assume that mobilisation of service providers through participation and their subsequent sense of ownership about the quick turnaround co-designed solutions will provoke some short term local improvements.

#### Creating an enabling environment for young women to exercise choices

In 2002 a large national survey of youth in schools in South Africa led to development of an audio-drama called *Beyond Victims and Villains* (BVV) based on the survey findings and aiming to stimulate discussion around gender violence and HIV risk (http://www.ciet.org/en/project/south-africa-education-on-sexual-violence-and-hiv-aids-beyond/). Updated with results of a 2012 survey in Botswana, the eight-episode audio-drama in INSTRUCT intervention districts will support community-wide structured discussions, aiming to generate local initiatives to reduce gender violence and to increase awareness of choice disability. INSTRUCT investigators will train key community members and service providers to facilitate BVV discussions: community activists, especially men active in their communities; health education assistants from the government clinics; guidance teachers from government primary and secondary schools; and traditional healers. The training, over three days, will cover facilitation skills as well as providing a thorough understanding of the contents of the audio-drama. Each trainee will receive an MP3 player with the audio-drama episodes on a memory stick and a facilitator’s guide.

The trained health education assistants, community members and traditional healers will implement discussions with different groups in the community, covering young and old, male and female. Guidance teachers will implement audio-drama and discussion sessions with school students. Typically, groups will discuss one episode a week. Discussion of each episode will end by asking the group what they and others in the community can do about the issue covered in the episode. Following each episode, the facilitator will encourage members of the group to talk to their families, friends and neighbours about the issues raised in the audio-drama.

### Encouraging participation

This project fosters authentic and meaningful participation of a broad range of stakeholders (including intermediaries and beneficiaries) throughout the project’s life cycle. In the communities where the key beneficiaries – young women out of work and out of education aged 15–29 – live, we will engage men in audio-drama groups run by traditional doctors, and older women will be engaged in the women’s groups. Boys will be engaged in schools alongside girls, as the BVV program is implemented in schools in the five districts. The young women targeted by the intervention will be active participants in the recruitment of other participants, in the identification of obstacles and opportunities for access to programs, and in the deliberative dialogue that co-designs solutions. Our work in Canada and elsewhere shows this type of participation changes the objective position and self-confidence of young women, in addition to identifying program fixes that are better suited to their needs [42–45]. Service providers are likewise participants in the identification of opportunities and obstacles during the fuzzy cognitive mapping and in the deliberative dialogue leading to co-design of quick turnaround and systemic fixes.

### Modifying the interventions and adherence to protocol

The INSTRUCT protocol is the same in all communities across the five intervention districts, but the details of how the intervention plays out will differ between communities and between districts, with local stakeholders determining exactly what they do and how they do it. This participatory approach to RCTs of interventions for public health issues is gaining momentum [46–49], as it allows contextualisation of and local buy-in to a standard intervention. A trial of community mobilisation for prevention of dengue in Mexico and Nicaragua that took this approach demonstrated a significant impact on entomological indices and on rates of serological dengue infection and clinical dengue illness [50–52].

In addition to following up young women after workshops, the district coordinators and INSTRUCT investigators will maintain frequent contact with government program officers and managers and with trained BVV facilitators. We expect a considerable turnover of trained community facilitators, health education assistants and teachers so that further trainings of audio-drama facilitators will be necessary. We will use social network analysis to examine the networks of young women for information, socialising and emotional support, and the way such networks might be used to reach young women with information – for example about government support programs – more effectively.

Recognizing that local context can affect the impact differently from district to district, we will carry out mid-stream intervention research that will map out these context factors using a mixture of written and oral narratives, key informant interviews, impact logs, and inter-organizational network analysis [53]. We will use the Most Significant Change technique to explore change dynamics and language [54]. The concern of this intervention research within INSTRUCT is how intervention effects are modified by context.

### Outcomes

The *primary outcome* is HIV prevalence in young women aged 15–29 years, measured in an impact survey in the five intervention and five control (second wave of intervention) districts. We will undertake rapid HIV screening testing, using Alere Determine units to test finger prick blood samples for HIV1 and HIV2 antibodies. We will not perform confirmatory testing for those that test reactive on this screening test. However, those reactive on the screening test will be referred to their local government clinic for confirmatory testing, counselling, and treatment if appropriate.

*Secondary outcomes* will rely on responses to a questionnaire for men and women aged 15–29 years administered in the impact survey in intervention and control districts. We will measure intermediate outcomes between knowledge and behaviour, according to the CASCADA behaviour change model [55, 56], based on the Theory of Planned Behaviour [57]. CASCADA is an acronym for a partial order of intermediates between knowledge and action: Conscious knowledge, Attitudes, Subjective norms, intention to Change, Agency to make the change, Discussion with family, peers and neighbours, and finally Action. The model does not assume that intermediate steps between conscious knowledge and action are necessarily linearly related. We have used the CASCADA model in resource-poor settings to support analysis and interpretation in cross-sectional studies examining health-related behaviours [58, 59], to guide design of interventions [50, 60, 61] and as a framework for qualitative and quantitative analysis of intervention effects [62, 63]. In this trial, the questionnaire will collect information on the CASCADA sequence of intermediate variables for use of government programs, engagement in transactional sex, gender violence, physical intimate partner violence, multiple partners, and condom use.

We will document costs of the intervention to estimate the cost per HIV case avoided. Monthly budgets and review of actual expenditure, for central and district activities, will provide the basis for documenting costs. In parallel, records of grants and loans made to marginalized young women by government structural support programmes will document this additional expenditure focused on HIV prevention. Estimated intervention costs will include pro-rated salary costs of government personnel who spend part of their time implementing the interventions (such as health education assistants and teachers facilitating BVV sessions). Salary and other costs of the central team activities will form a separate category as only a proportion of these costs will be incurred by the programme if it is successfully rolled-out more widely and longer-term.

### Participant timeline

**Fig. 1:**
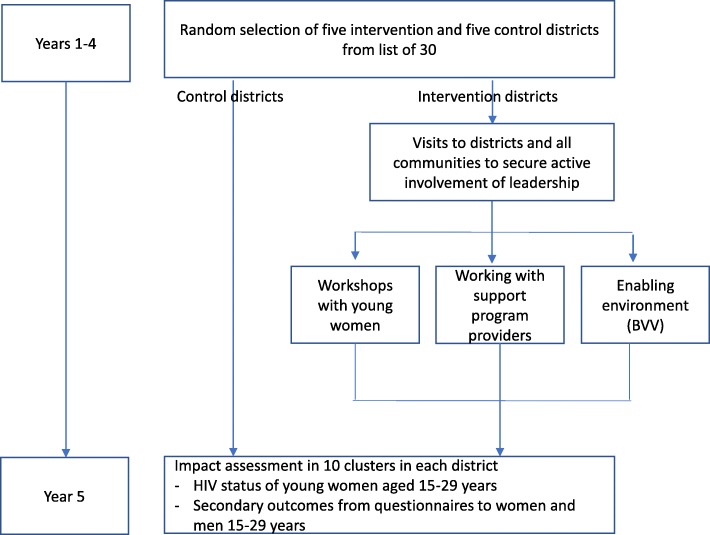
Schematic representation of the study design and timelines

The schedule of district allocation, interventions and impact assessment is shown in Fig 1. Enrolment in the first wave of five districts will begin in mid-2014 and continue to the end of 2018. We will begin the intervention in one of the five districts, extending to the other four in years 2–3 of the project.

### Sample size

The intervention package will potentially affect all young women in each intervention district. However, the impact assessment will rely on a cluster random sample of communities drawn from the recent census. There are thus two quite different domains – the domain of intervention (*all* young women in the intervention districts) and the domain of the survey (the *sample* of villages or clusters that will provide evidence of the impact on young women in the district) within which we will include all young women with no sub-sampling within the cluster.

Sample size calculations for the survey used a calculator developed by Taylor and colleagues [64]. The HIV prevalence in Botswana control clusters in the 2012 impact assessment was 23% among women aged 15–29 years (N Andersson, personal communication). In the Botswana AIDS Impact Survey of 2013, the HIV prevalence among women aged 15–49 years was 28.2% [39]. Since districts are the unit of randomization, intervention and analysis, starting from 23% (range 17–28%, k = 0.119), 650 women per district in five districts in each arm would detect a reduction to 17.25% (25% reduction in HIV) at the 5% significance level with 80% power. In the unpublished three-country study, the combined interventions in Botswana produced a reduction in HIV of around 50% (N Andersson, personal communication); these sample size calculations therefore allow for some inefficiency in taking the interventions to scale.

We will select a stratified random sample of 10 clusters (enumeration areas) from each of the five intervention districts, and 10 from the control (next wave of intervention) districts. From each cluster we will interview and test for HIV (rapid screening testing of finger prick blood) around 100 young people aged 15–29 years (at least 65 of them women). The sample will include around 5000 people from intervention districts and 5000 from non-intervention districts. To achieve these numbers, the overall sample size *invited to participate* in the survey will need to be around 7500 to allow for absentees and those who decline to participate. Some 13.1% of young women contacted in the 2012 survey in the three countries declined to participate, and a further 14% were absent at the times the interviewer could visit (N Andersson, personal communication).

Secondary outcomes (such as gender violence, unsafe sexual behaviours) are more common occurrences and the power to detect these is correspondingly greater.

### Allocation

Central randomization of districts will stratify by two factors (1) HIV prevalence (above or below the national average), using results of the fourth Botswana AIDS Impact Survey (BAIS IV) [39] and (2) development and economic status including proportions of urban and rural communities.

An epidemiologist not involved in the fieldwork will centrally randomize districts using an online random number generator, with allocation concealment until commencement of each wave. We will unmask the first wave districts immediately, and the second wave in year 5. Local intervention activities will be obvious to residents in the intervention sites. Knowledge of *future* intervention status may become clear to residents of the second wave at the time of their baseline study, in year 5. Some secondary outcomes (particularly conscious knowledge) could be influenced by knowledge of intervention status. Other indicators like a reduction in gender violence or HIV status would be less susceptible to this bias.

Interviewers in the impact survey will not include intervention fieldworkers and, as far as possible, they will be kept unaware if the communities in which they are interviewing are in intervention or non-intervention districts. The principal analysis will be undertaken blind of intervention status, using only group labels for each district.

### Data collection and analysis for impact measurement

For the impact survey, in the stratified random sample 10 clusters (enumeration areas) in each of the intervention and control districts, trained fieldworkers will conduct the interviews and take finger prick blood samples for rapid HIV screening testing. The interviewers will go house to house, starting from a random point, until they reach the target number of interviews; there will be no sub-sampling within the cluster.

The fieldworkers will administer the questionnaire in the Setswana language. The questionnaire will collect information about the secondary outcomes of use of government support programs, gender violence and sexual behaviours described above, including intermediate variables based on the CASCADA model of behaviour change. Specific questions will include those we have used and validated in previous surveys in the region in 2007 [65–67] and 2008 [68].

The interviewers will use Open Data Kit (ODK) Collect [69] on Android tablets to record responses. Field supervisors will check data on the tablets and upload the records to ODK Aggregate hosted on a server in Botswana.

The fieldworkers will take the finger prick blood samples in the household setting, using single-use safety lancets, with a blade that retracts immediately into the hub after use, to eliminate the theoretical risk of blood exposure while taking the sample.

#### Statistical methods

We will adopt an intention-to-treat principle for measuring primary outcomes with district as the unit of analysis. The primary analysis will treat the two groups of districts as a parallel design, and will use a two-sample t-test to compare the HIV prevalence rate of young women aged 15–29 years in the five first wave (intervention) and the five second wave (control) districts. Since the impact assessment in the first wave will coincide with the baseline in the second wave, the analysis will be able to ignore temporal effects.

Secondary analysis will compare HIV and gender violence related knowledge, attitudes and behaviours in young women and men between intervention and non-intervention districts, adjusting for clustering. Adjusted analysis using regression techniques will investigate the residual impact of key baseline characteristics on the outcomes. We will examine the residuals for model assumptions and chi-squared test of goodness-of-fit. Analysis will rely on CIETmap software [70] which provides a windows interface with the statistical programming language R.

We will summarize district demographics and outcome variables (both primary and secondary) using descriptive measures: mean (standard deviation) or range (minimum-maximum) for continuous variables and number (percent) for categorical variables.

Sensitivity analysis will examine correlated outcomes using generalized estimating equations, assuming intervention as a fixed effect, district as clustering variable and an exchangeable or unstructured covariance structure to model correlation of participants within a district. Planned subgroup analysis will focus on subgroups of young women with different exposures [71]. Age is a core issue in gender violence and HIV incidence. A history of sexual abuse is likely to affect responses across a range of themes. Variables on use of alcohol and other substances may be informative, as well as employment or educational status and exposure to workshops in the intervention districts.

We will use multiple-imputation to deal with missing data [72]. All statistical tests will be two-sided at the 0.05 level of significance. The Bonferroni method will adjust the level of significance for testing for secondary outcomes to keep the overall level at alpha = 0.05. We will express results as effect or odds ratio/relative risk reduction for binary outcomes, standard errors, corresponding two-sided 95% confidence intervals and associated *p*-values.

### Ethics

The trial received ethical approval from the Health Research and Development Committee (HRDC) in the Ministry of Health, Botswana, on 8 August 2013, HRDC protocol number 00724.

#### Informed consent

The research team will seek the agreement of the district leadership in districts randomized to receive the intervention, including the district commissioner, the head of the district health management team and the district AIDS coordinator. We will also seek agreement to participate from the leadership (the chairman of the village development committee and the village chief) in all communities in the intervention districts. In the process of cluster randomization of whole districts to the intervention package it will not be logistically feasible to obtain informed consent from all those individuals in the districts who might be exposed to interventions. The trial is about the impact of the *offer* of the intervention package: the decision to participate or not in any element of the package rests with the individual residents of the intervention districts.

For the impact survey, interviewers will seek informed consent from all respondents. They will explain the nature and purpose of the survey and its voluntary character. They will explain that participants may decline to answer any questions and may terminate the interview at any time. They will ask respondents for their consent to participate, including giving a finger prick blood sample for rapid screening HIV testing, and participants will sign to indicate their consent. For participants under age 16, a parent or guardian of the young person will sign to indicate their consent. The young person will also be asked to assent to participate.

#### Confidentiality

Training of fieldworkers will emphasise their responsibility for maintaining confidentiality of all information they have access to during the work. We will report only grouped findings, in a way that does not allow identification of any individuals or individual communities.

Fieldworkers will administer the electronic questionnaire in a secluded spot in the homestead, taking care to maintain privacy throughout the interview. The questionnaires will not record names and no individual will be identifiable in the database derived from the questionnaires.

Digital records will be kept securely on the server in Botswana and will be accessible only to the principal investigators and to those they explicitly delegate to assist in data analysis. Original paper consent forms, will be securely transported, stored, retained and finally destroyed in accordance with CIET guidelines for security, storage and eventual destruction of paper records, available on our website (www.cietresearch.org).

#### Minimising potential risks to participants

The survey and finger-prick blood sample do not present more than minimal risk to participants. Finger prick blood sampling involves minor discomfort. We will train fieldworkers thoroughly in the procedure for obtaining finger prick blood samples and undertaking the rapid testing procedure, and assess their competence before allowing them to work in the field teams. The field workers will refer participants who test positive on the rapid HIV screening test to the local government clinic for confirmatory testing, counselling, and treatment if appropriate.

### Knowledge translation and exchange

The project takes a participatory approach to knowledge translation, involving research users throughout the research process [73]. Government partners, young women and service providers in the intervention districts have already contributed to the research design. Initial design workshops with government counterparts will finalise design and implementation. Management meetings during the project with government counterparts will update them on progress, share problems and seek solutions. Interim results dissemination meetings with local stakeholders will share preliminary findings, and discuss any required modifications to the process.

As results become available at the end of the trial, dissemination workshops will share findings with government and non-government stakeholders in Botswana. The research team will present findings at conferences in Africa and elsewhere, where possible involving research users in this process, and publish articles on the research process and findings in peer reviewed journals.

## Discussion

The presently funded project is the first stage of a longer national process implementing an HIV structural prevention package in waves of five districts in a stepped-wedge design. If funding is available, we will start the intervention package in the second wave of districts once the impact survey in the first and second wave districts is completed (in year 5). The second wave intervention package will include modifications based on the experience during the first wave of implementation. With the Government of Botswana, we will seek funding for extension of the intervention and impact measurement from national and international sources. If the first and/or second waves of interventions are successful in reducing HIV infections, the government may decide to roll out the package in all remaining districts as rapidly as possible.

### Potential achievements related to the research

This project illustrates a health development approach that may be of wider relevance. It identifies choice disability leading to disproportionate HIV risk. The resulting intervention opens a dialogue about the nature of the exclusion with the communities and the services in the context of the structural shifts in favour of young women. Even if the structural shifts (for example, young women entrepreneurs with purchasing power and self esteem to make transactional trans-generational sex less worthwhile) affect relatively few women, the logic for inclusion of young women shifts the meaning of the “presumed average citizen” as the target for services. This shift to include the most vulnerable, without taking away services from other beneficiaries, effectively strengthens systems, increasing coverage and, potentially, effectiveness and efficiency.

Gender violence, distorted traditionalist views of gender hierarchy, gendered economic dependence and alcohol abuse all lead to choice disability in HIV prevention. Enabling choices shifts power balances and reduces the worst effects of the disdain for the safety of others that is often part of sexual violence and transactional sex. Importantly, by increasing the number who can choose existing prevention actions – such as abstention, condom use or reduced concurrency -- purposive enabling of choice will release the potential impact of other HIV prevention strategies. INSTRUCT will help fill in the gaps in the literature and move the field forward by documenting the effect that a package of combined structural and behavioural interventions has on HIV infection among the age group that matters most for HIV prevention.

### Risks and mitigation strategies

The project includes re-targeting government structural support programmes towards those most vulnerable to HIV. This involves intensive cooperation from the government bodies responsible for implementing these programmes. Although these bodies at national level have expressed support for the project in principle, it may be hard to translate into a different way of doing business in practice. Among other changes, this will require increased support for the most vulnerable programme beneficiaries and increased monitoring of the programmes.

Currently access to government structural support programmes is patchy and not ideal, even in terms of poverty alleviation, let alone HIV prevention. During the project we will work with government to develop more effective monitoring of the government programmes: What is the age, sex of the beneficiaries? What are the outcomes of grants and loans? We will also undertake intervention research to examine the implementation and processes of the intervention package. The risk of incomplete or ineffective implementation of program changes in favour of young women is a key challenge of INSTRUCT, and will be monitored and addressed throughout the project.

## Abbreviations

BVV: Beyond Victims and Villains audio-drama
CRCT: Cluster randomised controlled trial
DC: District Commissioner
FCM: Fuzzy cognitive mapping
FW: Focused workshop
HEA: Health education assistant
NACA: National AIDS Coordinating Agency
RCT: Randomised controlled trial
